# Dual Nature of RAGE in Host Reaction and Nurturing the Mother–Infant Bond

**DOI:** 10.3390/ijms23042086

**Published:** 2022-02-14

**Authors:** Yu Oshima, Ai Harashima, Seiichi Munesue, Kumi Kimura, Nontaphat Leerach, Hisanori Goto, Mariko Tanaka, Akane Niimura, Kenjiro Hayashi, Hiroshi Yamamoto, Haruhiro Higashida, Yasuhiko Yamamoto

**Affiliations:** 1 Department of Biochemistry and Molecular Vascular Biology, Kanazawa University Graduate School of Medical Sciences, Kanazawa 920-8640, Japan; cantabile.6102@gmail.com (Y.O.); aharashima@staff.kanazawa-u.ac.jp (A.H.); smunesue@med.kanazawa-u.ac.jp (S.M.); kukimura@staff.kanazawa-u.ac.jp (K.K.); nleerach@gmail.com (N.L.); pitanori19890838@gmail.com (H.G.); plaplastichq@med.kanazawa-u.ac.jp (M.T.); imymemine@stu.kanazawa-u.ac.jp (A.N.); ke.hayashi@po.nippon-shinyaku.co.jp (K.H.); hiroshi.yamamoto@komatsu-u.ac.jp (H.Y.); 2 Komatsu University, Komatsu 923-0921, Japan; 3 Department of Basic Research on Social Recognition and Memory, Research Center for Child Mental Development, Kanazawa University, Kanazawa 920-8640, Japan; haruhiro@med.kanazawa-u.ac.jp

**Keywords:** receptor for advanced glycation end-products (RAGE), oxytocin, blood–brain barrier, intestinal barrier, maternal bonding, social behavior

## Abstract

Non-enzymatic glycation is an unavoidable reaction that occurs across biological taxa. The final products of this irreversible reaction are called advanced glycation end-products (AGEs). The endogenously formed AGEs are known to be bioactive and detrimental to human health. Additionally, exogenous food-derived AGEs are debated to contribute to the development of aging and various diseases. Receptor for AGEs (RAGE) is widely known to elicit biological reactions. The binding of RAGE to other ligands (e.g., high mobility group box 1, S100 proteins, lipopolysaccharides, and amyloid-β) can result in pathological processes via the activation of intracellular RAGE signaling pathways, including inflammation, diabetes, aging, cancer growth, and metastasis. RAGE is now recognized as a pattern-recognition receptor. All mammals have RAGE homologs; however, other vertebrates, such as birds, amphibians, fish, and reptiles, do not have RAGE at the genomic level. This evidence from an evolutionary perspective allows us to understand why mammals require RAGE. In this review, we provide an overview of the scientific knowledge about the role of RAGE in physiological and pathological processes. In particular, we focus on (1) RAGE biology, (2) the role of RAGE in physiological and pathophysiological processes, (3) RAGE isoforms, including full-length membrane-bound RAGE (mRAGE), and the soluble forms of RAGE (sRAGE), which comprise endogenous secretory RAGE (esRAGE) and an ectodomain-shed form of RAGE, and (4) oxytocin transporters in the brain and intestine, which are important for maternal bonding and social behaviors.

## 1. Introduction

Glycation is a reaction in which biological macromolecules (proteins, lipids, and nucleic acids) and the excessive reducing sugars and their metabolic derivatives are combined, leading to alterations in their structures and functions in the body. Advanced glycation end products (AGEs) are a broad heterogeneous group of compounds formed by non-enzymatic reactions. The accumulation of endogenous and exogenous AGEs has been implicated in the pathogenesis of numerous diseases in humans [1,2]. Sustained hyperglycemia under diabetic conditions can lead to increased production of AGEs in vivo [1,2]. In addition, diet is an important exogenous source of AGEs and contributes to an in vivo AGE pool. It has been reported that approximately 10% of dietary AGEs are absorbed after oral ingestion and then assimilated into the circulation via the human gastrointestinal tract [3]. AGEs can induce intrinsic cell signaling pathways and, in turn, contribute to the development of various diseases via the receptor for AGEs (RAGE) on cell membranes [2,4,5].

Anti-aging treatments have attracted increasing attention in recent years, focusing on anti-glycation to reduce morbidity, ensure healthier aging and longevity, and promote cosmetic enhancement. Targeting RAGE could be a preventive and therapeutic strategy against various RAGE-associated diseases, including inflammatory disorders, diabetes mellitus and its complications, aging-related diseases, neurodegenerative disorders, and cancer growth and metastasis [2,4,5,6,7,8,9,10,11,12,13,14,15,16].

RAGE is a multiligand pattern-recognition receptor belonging to the immunoglobulin superfamily [4,5,6]. We recently discovered that RAGE present on brain vascular endothelial cells can bind oxytocin (OT) and transport it from the blood to the brain, resulting in the regulation of brain OT levels. Research on OT in the brain has attracted increasing attention, as the molecule plays an important role in social behaviors such as recognition, trust, anti-anxiety behavior, and mother–infant bonding [17,18]. This discovery of RAGE-mediated OT transport will open a new avenue for the link between energy metabolism, glycation, aging, and OT for brain function and social behaviors in mammals.

In this review, we highlight the recent progress made in understanding the role of RAGE in physiological and pathophysiological processes, including host defense responses, exaggerating host reactions, and social behaviors.

## 2. Glycation, AGEs and RAGE

Glycation is a non-enzymatic and unavoidable background reaction that occurs in all living beings and results in the formation of AGEs. Apart from AGEs, RAGE is known to interact with a series of different ligands, including high-mobility group box-1 (HMGB1), Gram-negative bacterial cell wall lipopolysaccharides (LPS), S100 proteins, complement component C3, phosphatidylserine (PS), and amyloid-β. The chemical structures of AGEs include *N*^ε^-carboxy-methyl-lysine (CML), *N*^ε^-carboxy-ethyl-lysine (CEL), glyceraldehyde-derived pyridinium (GLAP), glycolaldehyde (GA)-pyridine, pentosidine, and methylglyoxal-derived hydroimidazolone 1 (MG-H1) [2,5,8,16,19,20]. The CML-modified S100A8/A9 strongly activates intestinal inflammatory responses via RAGE, which suggests that complex varieties of RAGE ligands are modified by glycation reactions [21].

RAGE has an extracellular (V, C1, and C2 domains) region, a transmembrane region, and a short cytoplasmic tail (ctRAGE) of 43 amino acids with a high charge [2,5]. For signal transduction, ctRAGE required an adaptor protein, diaphanous-related formin 1 (Diaph1), which led to the phosphorylation of its downstream effector protein Rac1, an essential factor for cell movement in rat C6 glioma cells [22]. The ctRAGE/Diaph1 interaction could be a potential therapeutic target for RAGE-associated diseases [23,24]. Furthermore, the extracellular RAGE antagonists such as low molecular weight heparin (LMWH), azeliragon (TTP488), papaverine, *N*-Benzyl-4-chloro-*N*-cyclohexylbenzamide (FPS-ZM1), and RAGE-antagonist peptide (RAP) are also known to inhibit disease development [25,26,27,28,29,30].

## 3. Role of RAGE in Physiological and Pathological Processes

A growing body of evidence suggests that RAGE plays a significant role in pathological processes of disease development and progression, as well as in physiological functions, including host defense, tissue regeneration, clearance of apoptotic cells, and nurturing the mother–infant bond (Table 1). RAGE has been reported to contribute to inflammation and fibrosis in the lungs and livers of experimental animal models [12,19,31,32,33]. Vascular injury, inflammatory reactions, and delayed neuronal cell death were attenuated in RAGE-deficient mice after transient brain ischemia via bilateral common carotid artery occlusion (BCCAO) [34]. Traumatic brain injury was also found to be ameliorated in RAGE-deficient mice [35]. Furthermore, RAGE mediated the progression of Alzheimer’s disease via amyloid β-induced neurotoxicity [36]. With regard to lifestyle-related diseases, RAGE has been reported to accelerate chronic inflammation and foam cell formation during the pathogenesis of atherosclerosis, diabetic kidney dysfunction and glomerulosclerosis, and obesity and pancreatic β-cell damage in diabetes [6,7,9,10,25,37,38]. In the context of tumor malignancy, RAGE is associated with chronic inflammation-mediated carcinogenesis in the skin and tumor progression driven by non-tumor cells in the microenvironment [11,39,40]. The role of RAGE in bacterial infection, sepsis, and septic shock is still unclear; however, factors such as the species and number of bacteria, route of infection, and genetic background of the animal have been shown to affect host defense reactions [19,41,42,43,44,45]. Nonetheless, exaggerated host immune reactions can cause severe tissue damage and reduce life expectancy; adequate host defense responses would prevent the dissemination of the bacteria and enhance clearance of the bacteria and endotoxin. In terms of physiological function, RAGE was shown to attenuate adaptive inflammation in limb ischemia and kidney ischemia-reperfusion injury using aseptic experimental models [46,47]. In addition, it has been reported that HMGB1-dependent lung epithelial regeneration and repair occur through RAGE [48]. Furthermore, RAGE contributes to the clearance of apoptotic cells via the recognition of PS, that is, the “eat me signal” [8,49], and is involved in nurturing the mother–infant bond and behaviors. The details of the aforementioned effects are outlined in Table 1.

## 4. RAGE Isoforms

**Figure 1:**
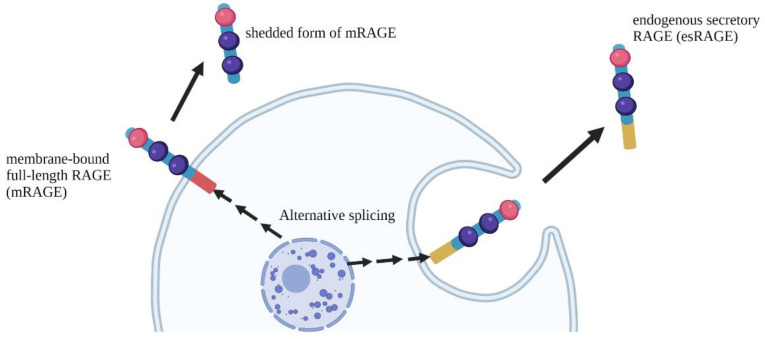
Schematic diagram of RAGE variants. Membrane-bound full-length RAGE (mRAGE) is the signal transduction form expressed on the cell surfaces. The soluble forms of RAGE (sRAGE) include endogenous secretory RAGE (esRAGE), a product of an alternatively spliced mRNA, and an ectodomain-shed form of mRAGE.

It is well known that RAGE has several isoforms (Figure 1). Membrane-bound full-length RAGE (mRAGE) is the active signal transduction form expressed on cell surfaces. Furthermore, the soluble forms of RAGE (sRAGE) include endogenous secretory RAGE (esRAGE), a product of an alternatively spliced mRNA, and an ectodomain-shed form of mRAGE [2,5,51,52,53,54]. sRAGE contains an extracellular domain that can bind to circulating pro-inflammatory ligands, preventing their binding to mRAGE, which, in turn, prevents RAGE activation as a decoy (Figure 1). Therefore, the balance between sRAGE and mRAGE is important for assessing morbidity risk and the development of pathophysiological conditions. It has been previously reported that RAGE deficiency (i.e., absence of mRAGE and sRAGE) and treatment with purified recombinant sRAGE in mice lead to a protective effect in organs under various pathological conditions, such as acute lung injury, diabetic atherosclerosis, kidney diseases, Alzheimer’s disease, and septic shock [2,5,19,55]. In contrast, we have recently shown that acute kidney disease in a renal ischemia reperfusion injury model is exacerbated under RAGE-deficient conditions, and hypoxic stress downregulates the expression of both mRAGE and sRAGE/esRAGE in renal tubular cells [47]. Furthermore, recombinant sRAGE administration has been reported to have a renoprotective effect against tubular injury in a renal ischemia reperfusion injury model [47].

## 5. RAGE and OT Nurtures the Mother–Infant Bonding

Genomic data indicate the existence of RAGE homologs in all mammals [56]. However, there are no RAGE homologs in other vertebrates, such as birds, amphibians, fish, and reptiles [56]. This evidence from an evolutionary perspective allows us to understand why mammals require RAGE and what its physiological roles are. One characteristic of all mammals is lactation, and all mammals secrete OT to stimulate nursing-associated milk letdown. OT is a neuropeptide synthesized primarily in the magnocellular neurons of the paraventricular and supraoptic nuclei of the hypothalamus. OT plays a prominent hormonal role in female reproduction, and its two primary peripheral effects are uterine contractions during childbirth and lactation during breastfeeding. The effects of OT range from the modulation of neuroendocrine reflexes to the fundamental roles of complex bonding and social behaviors related to the reproduction and care of offspring [5,18,57,58]. It is well known that OT produces a wide spectrum of central and peripheral effects. Practical nasal administration of large doses of OT has been attempted in humans with and without social deficit-related psychiatric disorders, such as autism spectrum disorders and schizophrenia [57,58]. Intranasal administration of OT is believed to be effective in the central delivery of OT across the blood–brain barrier (BBB) [57,58]. However, there is a dearth of direct evidence for this transport process. Our group demonstrated that mRAGE on endothelial cells of the BBB can bind OT and transport the neuropeptide from the blood into the brain, resulting in the regulation of brain OT levels [6,7]. OT cannot compete with the interaction of mRAGE with other ligands or induce mRAGE intracellular signaling [17,18]. In addition, we reported that OT transfer by mRAGE is unidirectional from the blood to the brain [17,18]. The expression of mRAGE was upregulated in the cerebrovascular endothelium after transient brain ischemia was induced via BCCAO in mice [17,34]. Using this BCCAO model, it was found that OT transport into the brain was enhanced [17].

Breast milk contains OT, which is also concentrated in the mother’s circulation. Although OT in breast milk can be absorbed into the blood of newborn babies without any damage or impairment to the digestive tract, it remains unclear whether OT is permeable after the onset of gut closure, whether it is indeed permeable, and whether OT absorption is a receptor-mediated process. Immediately after birth and before the formation of the intestinal barrier, OT permeates the intestinal epithelial cells relatively freely; however, after the formation of the intestinal barrier, mRAGE plays a role in transporting OT across the small intestine [50].

**Figure 2:**
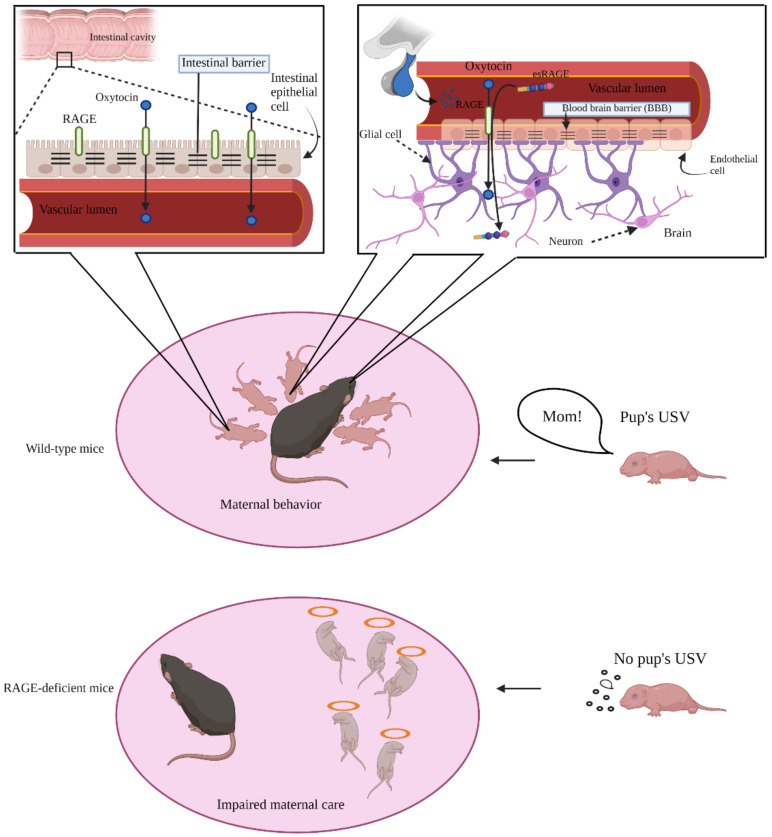
Schematic diagram of mRAGE as an oxytocin (OT) transporter in the intestinal barrier and the blood–brain barrier (BBB) for nurturing mother–infant bonding. The ultrasonic vocalization (USV) is an early communicative behavior between pup and mother.

We found that exogenously injected OT was not transported into the brain via the BBB in RAGE-deficient mice, and the mice showed impaired mother–infant bonding [6,7]. In other words, RAGE-deficient mother mice (dams) exhibited impaired parental care for their pups when exposed to environmental stress conditions, such as cage switching one day before delivery [17,18,59]. Anxiety-related behavior, parenting behavior of dams during pup retrieval, and ultrasonic vocalization (USV) measurement of mother-offspring pairing conditions were also examined. RAGE-deficient dams displayed anxiety-like behavior and hyperactivity during the early postpartum period [59] (Figure 2). In addition, we found that RAGE-deficient pups at postnatal day 3 exhibited insufficient and impaired USV as an early communicative behavior toward their mother [59] (Figure 2). These findings indicate that mRAGE-dependent OT recruitment to the brain is essential during the early postpartum period in dams, pups, and presumably, the puerperium in humans.

We wondered whether sRAGE affects the mRAGE-dependent transfer of OT from the blood into the brain. Interestingly, sRAGE did not inhibit OT transport, and sRAGE itself was transported into the brain through the BBB by endothelial mRAGE [60,61]. We assume that mRAGE may form an oligomer complex with sRAGE on endothelial cells and transcytose sRAGE from the blood to the brain [62]. As previously alluded, the expression of endothelial mRAGE could be upregulated in brain ischemia [17,34]. It is conceivable that endothelial mRAGE is a double-edged sword; mRAGE activation and its signal transduction can induce vascular inflammation, whereas mRAGE can transport sRAGE, a decoy receptor, and OT into the brain, possibly preventing neuronal damage [60,61].

## 6. Conclusions

The current understanding of the essence of glycation, AGEs, and RAGE variants in physiological and pathological processes is summarized herein. mRAGE is recognized as an OT transporter that nurtures the mother–infant bonding, as well as a pattern-recognition receptor for mediating host defense reactions, leading to inflammatory diseases under excessive and unchecked conditions. This discovery of mRAGE-mediated OT transport would lead to the development of new therapeutic strategies for mental disorders such as schizophrenia and reactive attachment conditions such as autism spectrum disorder. This might also contribute to solving growing social problems, such as child neglect and abuse.

## Figures and Tables

**Table 1:** Role of RAGE in physiological and pathological processes.

Role of RAGE in Exaggerating Host Reaction			
	Experimental Model	Relevant Findings	Ref
**Pathological Processes**	Lung injury and fibrosis [LPS, HDM, bleomycin, elastase]	Proinflammatory and fibrotic	[12,13,19,31,32]
	Liver fibrosis [CCl4]	Fibrotic	[33]
	Brain injury [ischemia, trauma]	Enhanced injury	[34,35]
	Alzheimer’s disease [Ab]	Ab-induced perturbation of neuronal function	[36]
	Atherosclerosis [*Ldlr*^−/−^, *Apoe*^−/−^]	Chronic inflammation and foam cell formation	[37,38]
	Kidney injury and fibrosis [diabetes]	Accerelated kidney injury and glomerulosclerosis	[6,7,25]
	Obesity and diabetes [HFD, *db/db*]	Adipocyte heypertropgy, obesity and pancreatic b cell failure	[9,10]
	Carcinogenesis [DMBA/TPA]	Chronic inflammation and carcinogenesis	[39]
	Tumor microenviornment [glioma, breast cancer]	Non-tumor cells of the microenviornment drive tumor progression	[11,40]
	Infection [*S. pneumoniae*, *L. monocytogenes*]	Deleterious during bacterial inefection, but still unclear	[41,42]
	Sepsis and septic shock [LPS, CLP]	Severe inflammation	[19,43,44]
**Role of RAGE in Host Defense**			
**Physiology**	**Experimental Model**	**Relevant Findings**	**Ref**
	Infection [*K. pneumoniae*]	Prevention of the dissemination	[45]
	Limb ischemia [femoral artery ligation]	Attenuation of adaptive inflammation	[46]
	Kidney reperfusion injury [ischemia reperfusion]	Protection by endogenous soluble RAGE	[47]
	Lung regeneration [HDM]	HMGB1-dependent epethelial repair	[48]
	Efferocytosis	Recognition of phosphatidylserine on apoptotic cells	[8,49]
**Role of RAGE in Nurturing the Mother-Infant Bond**			
**Physiology**	**Experimental Model**	**Relevant Findings**	**Ref**
	Parenting and affection [stress]	Oxytocin transfer from the blood to the brain via BBB and baby survival	[17]
	Oxytocin absorption	RAGE-dependent oxytocin transport in the small intestine	[50]

*Apoe*, apolipoprotein E; BBB, blood-brain barrier; BMBA/TPA, 7,12-dimetylbenz[a]anthracene/12-O-tetradecanoylphorbol-13-acetate; CCl4, carbon tetrachloride; CLP, cecal ligation and puncture; HDM, house dust mite; HFD, high fat diet; HMGB1, high mobility group box 1; *Ldlr*, low density lipoprotein receptor; LPS, lipopolysaccharides.

## Data Availability

Not applicable.

## References

[1] Rabbani N., Thornalley P.J. (2021). Protein glycation—Biomarkers of metabolic dysfunction and early-stage decline in health in the era of precision medicine. Redox Biol..

[2] Yamamoto Y., Yamamoto H. (2012). Controlling the receptor for advanced glycation end-products to conquer diabetic vascular complications. J. Diabetes Investig..

[3] Koschinsky T., He C.J., Mitsuhashi T., Bucala R., Liu C., Buenting C., Heitmann K., Vlassara H. (1997). Orally absorbed reactive glycation products (glycotoxins): An environmental risk factor in diabetic nephropathy. Proc. Natl. Acad. Sci. USA.

[4] Schmidt A.M., Vianna M., Gerlach M., Brett J., Ryan J., Kao J., Esposito C., Hegarty H., Hurley W., Clauss M. (1992). Isolation and characterization of two binding proteins for advanced glycosylation end products from bovine lung which are present on the endothelial cell surface. J. Biol. Chem..

[5] Leerach N., Harashima A., Munesue S., Kimura K., Oshima Y., Goto H., Yamamoto H., Higashida H., Yamamoto Y. (2021). Glycation reaction and the role of the receptor for advanced glycation end-products in immunity and social behavior. Glycoconj. J..

[6] Yamamoto Y., Kato I., Doi T., Yonekura H., Ohashi S., Takeuchi M., Watanabe T., Yamagishi S., Sakurai S., Takasawa S. (2001). Development and prevention of advanced diabetic nephropathy in RAGE-overexpressing mice. J. Clin. Investig..

[7] Inagi R., Yamamoto Y., Nangaku M., Usuda N., Okamato H., Kurokawa K., van Ypersele de Strihou C., Yamamoto H., Miyata T. (2006). A severe diabetic nephropathy model with early development of nodule-like lesions induced by megsin overexpression in RAGE/iNOS transgenic mice. Diabetes.

[8] He M., Kubo H., Morimoto K., Fujino N., Suzuki T., Takahasi T., Yamada M., Yamaya M., Maekawa T., Yamamoto Y. (2011). Receptor for advanced glycation end products binds to phosphatidylserine and assists in the clearance of apoptotic cells. EMBO Rep..

[9] Monden M., Koyama H., Otsuka Y., Morioka T., Mori K., Shoji T., Mima Y., Motoyama K., Fukumoto S., Shioi A. (2013). Receptor for advanced glycation end products regulates adipocyte hypertrophy and insulin sensitivity in mice: Involvement of Toll-like receptor 2. Diabetes.

[10] Han D., Yamamoto Y., Munesue S., Motoyoshi S., Saito H., Win M.T.T., Watanabe T., Tsuneyama K., Yamamoto H. (2013). Induction of receptor for advanced glycation end products by insufficient leptin action triggers pancreatic β-cell failure in type 2 diabetes. Genes Cells.

[11] Chen X., Zhang L., Zhang I.Y., Liang J., Wang H., Ouyang M., Wu S., da Fonseca A.C.C., Weng L., Yamamoto Y. (2014). RAGE expression in tumor-associated macrophages promotes angiogenesis in glioma. Cancer Res..

[12] Ullah M.A., Loh Z., Gan W.J., Zhang V., Yang H., Li J.H., Yamamoto Y., Schmidt A.M., Armour C.L., Hughes J.M. (2014). Receptor for advanced glycation end products and its ligand high-mobility group box-1 mediate allergic airway sensitization and airway inflammation. J. Allergy Clin. Immunol..

[13] Waseda K., Miyahara N., Taniguchi A., Kurimoto E., Ikeda G., Koga H., Fujii U., Yamamoto Y., Gelfand E.W., Yamamoto H. (2015). Emphysema requires the receptor for advanced glycation end-products triggering on structural cells. Am. J. Respir. Cell Mol. Biol..

[14] Tsubokawa D., Kikuchi T., Lee J.M., Kusakabe T., Yamamoto Y., Maruyama H. (2021). Venestatin from parasitic helminths interferes with receptor for advanced glycation end products (RAGE)-mediated immune responses to promote larval migration. PLoS Pathog..

[15] Anisuzzaman, Hatta T., Miyoshi T., Matsubayashi M., Islam M.K., Alim M.A., Anas M.A., Hasan M.M., Matsumoto Y., Yamamoto Y. (2014). Longistatin in tick saliva blocks advanced glycation end-product receptor activation. J. Clin. Investig..

[16] Sakatani S., Seto-Ohshima A., Shinohara Y., Yamamoto Y., Yamamoto H., Itohara S., Hirase H. (2008). Neural-activity-dependent release of S100B from astrocytes enhances kainate-induced gamma oscillations in vivo. J. Neurosci..

[17] Yamamoto Y., Liang M., Munesue S., Deguchi K., Harashima A., Furuhara K., Yuhi T., Zhong J., Akther S., Goto H. (2019). Vascular RAGE transports oxytocin into the brain to elicit its maternal bonding behaviour in mice. Commun. Biol..

[18] Yamamoto Y., Higashida H. (2020). RAGE regulates oxytocin transport into the brain. Commun. Biol..

[19] Yamamoto Y., Harashima A., Saito H., Tsuneyama K., Munesue S., Han D., Watanabe T., Asano M., Takasawa S., Okamoto H. (2011). Septic shock is associated with receptor for advanced glycation endproducts (RAGE) ligation of LPS. J. Immunol..

[20] Murakami Y., Fujino T., Hasegawa T., Kurachi R., Miura A., Daikoh T., Usui T., Hayase F., Watanabe H. (2018). Receptor for advanced glycation end products (RAGE)-mediated cytotoxicity of 3-hydroxypyridinium derivatives. Biosci. Biotechnol. Biochem..

[21] Andrassy M., Igwe J., Autschbach F., Volz C., Remppis A., Neurath M.F., Schleicher E., Humpert P.M., Wendt T., Liliensiek B. (2006). Posttranslationally modified proteins as mediators of sustained intestinal inflammation. Am. J. Pathol..

[22] Hudson B.I., Kalea A.Z., Del Mar Arriero M., Harja E., Boulanger E., D’Agati V., Schmidt A.M. (2008). Interaction of the RAGE cytoplasmic domain with diaphanous–1 is required for ligand–stimulated cellular migration through activation of Rac1 and Cdc42. J. Biol. Chem..

[23] Manigrasso M.B., Pan J., Rai V., Zhang J., Reverdatto S., Quadri N., DeVita R.J., Ramasamy R., Shekhtman A., Schmidt A.M. (2016). Small molecule inhibition of ligand-stimulated RAGE-DIAPH1 signal transduction. Sci. Rep..

[24] Leerach N., Munesue S., Harashima A., Kimura K., Oshima Y., Kawano S., Tanaka M., Niimura A., Sakulsak N., Yamamoto H. (2021). RAGE signaling antagonist suppresses mouse macrophage foam cell formation. Biochem. Biophys. Res. Commun..

[25] Myint K.M., Yamamoto Y., Doi T., Kato I., Harashima A., Yonekura H., Watanabe T., Shinohara H., Takeuchi M., Tsuneyama K. (2006). RAGE control of diabetic nephropathy in a mouse model: Effects of RAGE gene disruption and administration of low-molecular weight heparin. Diabetes.

[26] Takeuchi A., Yamamoto Y., Munesue S., Harashima A., Watanabe T., Yonekura H., Yamamoto H., Tsuchiya H. (2013). Low molecular weight heparin suppresses receptor for advanced glycation end products-mediated expression of malignant phenotype in human fibrosarcoma cells. Cancer Sci..

[27] Burstein A.H., Grimes I., Galasko D.R., Aisen P.S., Sabbagh M., Mjalli A.M. (2014). Effect of TTP488 in patients with mild to moderate Alzheimer’s disease. BMC Neurol..

[28] Deane R., Singh I., Sagare A.P., Bell R.D., Ross N.T., LaRue B., Love R., Perry S., Paquette N., Deane R.J. (2012). A multimodal RAGE-specific inhibitor reduces amyloid beta-mediated brain disorder in a mouse model of Alzheimer disease. J. Clin. Investig..

[29] El-Far A.H.A.M., Munesue S., Harashima A., Sato A., Shindo M., Nakajima S., Inada M., Tanaka M., Takeuchi A., Tsuchiya H. (2018). In vitro anticancer effects of a RAGE inhibitor discovered using a structure-based drug design system. Oncol Lett..

[30] Arumugam T., Ramachandran V., Gomez S.B., Schmidt A.M., Logsdon C.D. (2012). S100P-derived RAGE antagonistic peptide reduces tumor growth and metastasis. Clin. Cancer Res..

[31] Wang H., Wang T., Yuan Z., Cao Y., Zhou Y., He J., Shen Y., Zeng N., Dai L., Wen F. (2018). Role of receptor for advanced glycation end products in regulating lung fluid balance in lipopolysaccharide-induced acute lung injury and infection-related acute respiratory distress syndrome. Shock.

[32] He M., Kubo H., Ishizawa K., Hegab A.E., Yamamoto Y., Yamamoto H., Yamaya M. (2007). The role of the receptor for advanced glycation end-products in lung fibrosis. Am. J. Physiol. Lung Cell. Mol. Physiol..

[33] Ge X., Arriazu E., Magdaleno F., Antoine D.J., Dela Cruz R., Theise N., Nieto N. (2018). High mobility group box-1 drives fibrosis progression signaling via the receptor for advanced glycation end products in mice. Hepatology.

[34] Kamide T., Kitao Y., Takeichi T., Okada A., Mohri H., Schmidt A.M., Kawano T., Munesue S., Yamamoto Y., Yamamoto H. (2012). RAGE mediates vascular injury and inflammation after global cerebral ischemia. Neurochem. Int..

[35] Okuma Y., Liu K., Wake H., Zhang J., Maruo T., Date I., Yoshino T., Ohtsuka A., Otani N., Tomura S. (2012). Anti-high mobility group box-1 antibody therapy for traumatic brain injury. Ann. Neurol..

[36] Yan S.D., Chen X., Fu J., Chen M., Zhu H., Roher A., Slattery T., Zhao L., Nagashima M., Morser J. (1996). RAGE and amyloid-beta peptide neurotoxicity in Alzheimer’s disease. Nature.

[37] Soro-Paavonen A., Watson A.M., Li J., Paavonen K., Koitka A., Calkin A.C., Barit D., Coughlan M.T., Drew B.G., Lancaster G.I. (2008). Receptor for advanced glycation end products (RAGE) deficiency attenuates the development of atherosclerosis in diabetes. Diabetes.

[38] Sun L., Ishida T., Yasuda T., Kojima Y., Honjo T., Yamamoto Y., Yamamoto H., Ishibashi S., Hirata K., Hayashi Y. (2009). RAGE mediates oxidized LDL-induced pro-inflammatory effects and atherosclerosis in non-diabetic LDL receptor-deficient mice. Cardiovasc. Res..

[39] Gebhardt C., Riehl A., Durchdewald M., Németh J., Fürstenberger G., Müller-Decker K., Enk A., Arnold B., Bierhaus A., Nawroth P.P. (2008). RAGE signaling sustains inflammation and promotes tumor development. J. Exp. Med..

[40] Kwak T., Drews-Elger K., Ergonul A., Miller P.C., Braley A., Hwang G.H., Zhao D., Besser A., Yamamoto Y., Yamamoto H. (2017). Targeting of RAGE-ligand signaling impairs breast cancer cell invasion and metastasis. Oncogene.

[41] van Zoelen M.A., Schouten M., de Vos A.F., Florquin S., Meijers J.C., Nawroth P.P., Bierhaus A., van der Poll T. (2009). The receptor for advanced glycation end products impairs host defense in pneumococcal pneumonia. J. Immunol..

[42] Lutterloh E.C., Opal S.M., Pittman D.D., Keith J.C., Tan X.Y., Clancy B.M., Palmer H., Milarski K., Sun Y., Palardy J.E. (2007). Inhibition of the RAGE products increases survival in experimental models of severe sepsis and systemic infection. Crit. Care.

[43] Liliensiek B., Weigand M.A., Bierhaus A., Nicklas W., Kasper M., Hofer S., Plachky J., Gröne H.J., Kurschus F.C., Schmidt A.M. (2004). Receptor for advanced glycation end products (RAGE) regulates sepsis but not the adaptive immune response. J. Clin. Investig..

[44] Deng M., Tang Y., Li W., Wang X., Zhang R., Zhang X., Zhao X., Liu J., Tang C., Liu Z. (2018). The endotoxin delivery protein HMGB1 mediates caspase-11-dependent lethality in sepsis. Immunity.

[45] Achouiti A., de Vos A.F., van ‘t Veer C., Florquin S., Tanck M.W., Nawroth P.P., Bierhaus A., van der Poll T., van Zoelen M.A. (2016). Receptor for advanced glycation end products (RAGE) serves a protective role during *Klebsiella* pneumoniae-induced pneumonia. PLoS ONE.

[46] López-Díez R., Shen X., Daffu G., Khursheed M., Hu J., Song F., Rosario R., Xu Y., Li Q., Xi X. (2017). *Ager* deletion enhances ischemic muscle inflammation, angiogenesis, and blood flow recovery in diabetic mice. Arterioscler. Thromb. Vasc. Biol..

[47] Miyagawa T., Iwata Y., Oshima M., Ogura H., Sato K., Nakagawa S., Yamamura Y., Kamikawa Y., Miyake T., Kitajima S. (2021). Soluble receptor for advanced glycation end products protects from ischemia- and reperfusion-induced acute kidney injury. Biol. Open.

[48] Ojo O.O., Ryu M.H., Jha A., Unruh H., Halayko A.J. (2015). High-mobility group box 1 promotes extracellular matrix synthesis and wound repair in human bronchial epithelial cells. Am. J. Physiol. Lung Cell. Mol. Physiol..

[49] Friggeri A., Banerjee S., Biswas S., de Freitas A., Liu G., Bierhaus A., Abraham E. (2011). Participation of the receptor for advanced glycation end products in efferocytosis. J. Immunol..

[50] Higashida H., Furuhara K., Yamauchi A.M., Deguchi K., Harashima A., Munesue S., Lopatina O., Gerasimenko M., Salmina A.B., Zhang J.S. (2017). Intestinal transepithelial permeability of oxytocin into the blood is dependent on the receptor for advanced glycation end products in mice. Sci. Rep..

[51] Yonekura H., Yamamoto Y., Sakurai S., Petrova R.G., Abedin M.J., Li H., Yasui K., Takeuchi M., Makita Z., Takasawa S. (2003). Novel splice variants of the receptor for advanced glycation end-products expressed in human vascular endothelial cells and pericytes, and their putative roles in diabetes-induced vascular injury. Biochem. J..

[52] Harashima A., Yamamoto Y., Cheng C., Tsuneyama K., Myint K.M., Takeuchi A., Yoshimura K., Li H., Watanabe T., Takasawa S. (2006). Identification of mouse orthologue of endogenous secretory receptor for advanced glycation end-products: Structure, function and expression. Biochem. J..

[53] Motoyoshi S., Yamamoto Y., Munesue S., Igawa H., Harashima A., Saito H., Han D., Watanabe T., Sato H., Yamamoto H. (2014). cAMP ameliorates inflammation by modulation of macrophage receptor for advanced glycation end-products. Biochem. J..

[54] Cheng C., Tsuneyama K., Kominami R., Shinohara H., Sakurai S., Yonekura H., Watanabe T., Takano Y., Yamamoto H., Yamamoto Y. (2005). Expression profiling of endogenous secretory receptor for advanced glycation end products in human organs. Mod. Pathol..

[55] Egaña-Gorroño L., López-Díez R., Yepuri G., Ramirez L.S., Reverdatto S., Gugger P.F., Shekhtman A., Ramasamy R., Schmidt A.M. (2020). Receptor for advanced glycation end products (RAGE) and mechanisms and therapeutic opportunities in diabetes and cardiovascular disease: Insights from human subjects and animal models. Front. Cardiovasc. Med..

[56] Szwergold B.S., Miller C.B. (2014). Potential of birds to serve as a pathology-free model of type 2 diabetes, Part 1: Is the apparent absence of the rage gene a factor in the resistance of avian organisms to chronic hyperglycemia?. Rejuvenation Res..

[57] Jin D., Liu H.X., Hirai H., Torashima T., Nagai T., Lopatina O., Shnayder N.A., Yamada K., Noda M., Seike T. (2007). CD38 is critical for social behaviour by regulating oxytocin secretion. Nature.

[58] Lopatina O., Inzhutova A., Salmina A.B., Higashida H. (2013). The roles of oxytocin and CD38 in social or parental behaviors. Front. Neurosci..

[59] Gerasimenko M., Lopatina O., Munesue S., Harashima A., Yokoyama S., Yamamoto Y., Higashida H. (2021). Receptor for advanced glycation end-products (RAGE) plays a critical role in retrieval behavior of mother mice at early postpartum. Physiol. Behav..

[60] Munesue S., Liang M., Harashima A., Zhong J., Furuhara K., Boitsova E.B., Cherepanov S.M., Gerasimenko M., Yuhi T., Yamamoto Y. (2021). Transport of oxytocin to the brain after peripheral administration by membrane-bound or soluble forms of receptors for advanced glycation end-products. J. Neuroendocrinol..

[61] Shimizu Y., Harashima A., Munesue S., Oishi M., Hattori T., Hori O., Kitao Y., Yamamoto H., Leerach N., Nakada M. (2020). Neuroprotective effects of endogenous secretory receptor for advanced glycation end-products in brain ischemia. Aging Dis..

[62] Xue J., Manigrasso M., Scalabrin M., Rai V., Reverdatto S., Burz D.S., Fabris D., Schmidt A.M., Shekhtman A. (2016). Change in the molecular dimension of a RAGE-ligand complex triggers RAGE signaling. Structure.

